# Establishing Public Health Benchmarks for Physical Activity Programs

**Published:** 2006-06-15

**Authors:** Sarah Levin Martin, Tammy Vehige

**Affiliations:** Division of Nutrition and Physical Activity, Physical Activity and Health Branch, Centers for Disease Control and Prevention, Atlanta, Ga; Division of Nutrition and Physical Activity, Physical Activity and Health Branch, Centers for Disease Control and Prevention, Atlanta, Ga

## To the Editor:

Physical inactivity is associated with an increased risk for many chronic diseases, and the numerous health benefits of regular physical activity are well documented (1). Physical activity is ranked as the leading health indicator in *Healthy People 2010* (2).

State health departments have begun to address the burden of physical inactivity. At this early stage, it is imperative that sound training and technical assistance be provided. In 2003, the Physical Activity and Health Branch (PAHB) in the Division of Nutrition and Physical Activity at the Centers for Disease Control and Prevention (CDC) established five benchmarks outlining the areas of training and technical assistance that state health departments need to address to improve their capacity to promote physical activity to the public.

Physical activity professionals on the Research Application Team in PAHB convened to determine indicators of capacity needed to execute and manage community-based interventions that promote physical activity. Discussion began with the objectives of the Physical Activity and Public Health Practitioner's Course on Community Interventions, a 6-day training course developed by the University of South Carolina's Prevention Research Center in collaboration with and with funding from PAHB. The course objectives have since been refined by the two collaborating partners. Based on these objectives, PAHB established the following four benchmarks:

**1. Develop and sustain community partnerships.** By partnering with traditional and nontraditional partners, health departments can address the multiple levels of influence on physical activity, as suggested in the social–ecological model (3). This benchmark helps address the current status of the health field — being asked to do more with less (4) — because partnerships have great potential to leverage, combine, and capitalize on the complementary strengths of the entities involved.

**2. Use public health data as a tool to develop and prioritize community-based interventions**. This benchmark is based primarily on the concept of evidence-based practice, or data-driven decision making (5). Using available data — or better yet, collecting local data — aids strategic planning and allows progress to be tracked. Furthermore, achieving this benchmark may help eliminate disparities, which is a goal of *Healthy People 2010* (2).

**3. Understand and implement a sound approach to planning and evaluation.** This benchmark is also in line with evidence-based practice (5), because planning incorporates elements of the second benchmark (i.e., using data to target populations and develop interventions); in addition, carefully planned evaluation can yield new evidence. Sound program evaluation also engages stakeholders and is a useful tool for continuous quality improvement. Planning and evaluation are key aspects of health education and promotion in general.

**4. Implement evidence-based strategies at the 1) informational, 2) behavioral and social, and 3) environmental and policy levels.** This benchmark is based on the *Guide to Community Preventive Services*, which identified recommended strategies within these three domains based on existing scientific information (6).

The Research Application Team created the following fifth benchmark, which is not a part of the Physical Activity and Public Health Practitioner's Course:

**5. Develop an organizational structure that contributes to program growth and sustainability by encouraging and supporting professional development and fostering successful collaborations within and outside the health department.** Experience has taught the team that a supportive organizational structure is necessary to lead to a crosscutting physical activity program run by highly skilled physical activity practitioners.

CDC currently helps states address physical activity issues through a cooperative agreement with 28 states under the National Nutrition and Physical Activity Program to Prevent Obesity and Other Chronic Diseases. These states have a vested interest in promoting physical activity; they employ staff devoted to promoting physical activity and have completed or are finalizing state plans that include physical activity. Most have upper-management support and an organizational structure that encourages physical activity. These states receive regular technical assistance from CDC, and their staff attends mandated training. CDC also provides technical assistance to unfunded states through regular correspondence (by e-mail and telephone) to help them achieve the physical activity benchmarks and thereby increase their capacity for successful programs that promote physical activity to the public.

Given the impact of physical inactivity on the nation's health, CDC is working toward standardizing and enhancing the states' capacity to promote physical activity.

Providing technical assistance and training to state physical activity practitioners, either directly or through other government and nongovernment organizations that fund them, are among the highest priorities of PAHB's Research Application Team. The Research Application Team is using the physical activity benchmarks to focus the technical assistance it provides, and it is developing tools and resources to help states achieve them.
